# Stabilization of sunflower oil with pussy willow (*Salix aegyptiaca*) extract and essential oil

**DOI:** 10.1002/fsn3.389

**Published:** 2016-05-25

**Authors:** Zahra Sayyari, Reza Farahmandfar

**Affiliations:** ^1^ Department of Food Science and Technology, Savadkooh branchIslamic Azad UniversitySavadkoohIran; ^2^ Department of Food Science and TechnologySari Agricultural Sciences & Natural Resources University (SANRU)SariIran

**Keywords:** antioxidant activity, oxidative stability, pussy willow, sunflower oil, total phenolic content

## Abstract

The aim of present study was to evaluate antioxidant efficacy of pussy willow extract (PWE) and essential oil (PWEO) in stabilization of sunflower oil (SFO) during ambient storage (60 days at 25°C). Initially, total phenolic (TP) and total flavonoid (TF) contents were evaluated. Then, PWE, PWEO, and TBHQ were added to SFO. Peroxide value (PV), carbonyl value (CV), total polar compound (TPC), acid value (AV), and Oxidative stability index (OSI) were measured every 15 days. The results showed that PWE had higher TP and TF than PWEO (TP: 966.72 mg GAE/g and 355.8472 mg GAE/g, respectively; TF: 619.45 mg/100 g and 195.45 mg/100 g, respectively). Furthermore, according to all stabilization parameters, PWE had higher antioxidant efficacy followed by TBHQ, PWEO, and control, respectively. Therefore, PWE has antioxidant activity and it may be recommended as natural strong antioxidants to suppress lipid oxidation.

## Introduction

Sunflower oil (SFO) is an edible oil has been widely used due to its nutritional and medicinal value. sunflower oil is one source of linoleic acid ( 9 *cis*, 12 *cis* oxtadecadienoic) which has so many health beneficial effects (Hashemi et al. 2015). Autoxidation or oxidative rancidity is the major cause of quality losses in crude as well as refined oils during storage (Asnaashari et al. 2014a,b). When the crude and refined oils are exposed to factors such as heat, high temperatures, light, trace metals, or oxygen, the problem get more exacerbated (Crapiste et al. 1999; Sikwese and Duodu 2007; Farahmandfar et al. 2015a).

The use of antioxidants is the most appropriate way of stabilizing oils, preventing the autoxidation or oxidative rancidity, and protecting oils from the damages inflicted by free radicals (Farhoosh et al. 2016). The addition of synthetic antioxidants has been one of the most effective and popular method to prevent oxidative rancidity and development of off‐flavors (Eshghi et al. 2014). Investigations have demonstrated that synthetic antioxidants are toxic and carcinogenic for humans (Iqbal et al. 2008). Recently, there has been increased interest in identifying potential sources in order to obtain natural antioxidants (Poiana 2012; Farahmandfar et al. 2015b). Essential oil (EO) and extracts from spices, fruits, herbs, and hull contain effective compounds that prevent from the undesirable oxidative process in foodstuff (Asnaashari et al. 2015a).

*Salix aegyptiaca* L. (pussy willow) is generally cultivated in some provinces of Iran for hedge and ornamental purposes. The distillate obtained from male inflorescences of plants, with the common local name of “Araghe Bidmeshk” and English name of “Egyptian willow distillate,” in most parts of Iran have long been applied in folk medicine as cardiotonic (Sonboli et al. 2010). The aqueous extract and EO of *S. aegyptiaca* L. are being used in confectionery and flavorful syrups. In Iranian traditional medicine, *S. aegyptiaca* has been employed as laxative, cardioprotective, sedative, hypnotic, somnolent, aphrodisiac, orexigenic, carminative, gastroprotectant, anthelmintic, and vermifuge (Karimi et al. 2011). A number of chemical constituents such as flavonoids and volatile substances have been isolated from different parts of the plant (Enayat and Banerjee 2009; Karimi et al. 2011). From current pharmaceutical studies, additional pharmaceutical applications of *S. aegyptiaca* have revealed antioxidant, anti‐inflammatory, analgesic, anxiolytic, and antihypercholesterolemic effects among others (Hifnawy et al. 2010; Sonboli et al. 2010; Karimi et al. 2011; Rabbani et al. 2011).

Although previous studies have presented the antioxidant properties of pussy willow (PW) extract and EO, there are no reports on the antioxidant efficacy for the stabilization of SFO been shown so far. The purpose of this study was to estimate the antioxidant efficacy of PW flower extract and EO against oil oxidative rancidity and to compare their antioxidant activity with commercially available antioxidant (TBHQ).

## Materials and Methods

### Materials

Refined, bleached, deodorized SFO without any synthetic antioxidants was obtained from Ghoncheh Co. (Sari, Iran). The flowers of PW were purchased from the Ghamsar botanical garden at Kashan, Iran, during March 2014, and authenticated by the Medicinal Plant Incubator Center (Islamic Azad University, Ayatollah Amoli Branch, Amol, Iran). They were washed, air dried in the shade, and then powdered utilizing an electric device and stored in refrigerator (4°C) until use. TBHQ was prepared and purchased from Sigma‐Aldrich (St. Louis, MO). All other chemicals were of analytical grade and purchased from Merck Co., Frankfurt, Germany.

### Preparation of PWE and PWEO

The solvent (ethanol) was added to the powdered PW in the ratio of 10:1 (w:v) and the resulting mixture was shaken overnight. After 24 h, for separating the PW particles, the extract was filtered through Whatman No. 42 filter paper. The solvent was completely evaporated in an oven at 40°C. Finally, the obtained extract was stored in a dark container in refrigerator (4°C) until use (Esmaeilzadeh Kenari et al. 2014). EOs were extracted by hydrodistillation from the powdered PW by the Clevenger‐type apparatus, and the obtained oil was stored in a dark container in refrigerator (4°C) until use.

### Total phenolic content

The total phenolic (TP) content of PW extracts (PWE) and PWEO was determined according to the Folin–Ciocalteu reagent as described by Pourmorad et al. (2006) and the results were expressed as mg/g gallic acid equivalents (GAE). At a concentration of 1 mg/mL, PWE and PWEO were prepared in their own solvents, and 0.5 mL of each sample was mixed with 2.5 mL of a 10‐fold diluted Folin–Ciocalteu reagent and 2 mL of 7.5% sodium carbonate solution. After the samples were kept for 30 min at room temperature, the absorbance was measured at 760 nm in a UV‐Vis spectrophotometer (PG Instrument, T80, London, England).

### Determination of total flavonoid content

The total flavonoid (TF) content of PWE and PWEO was quantified according to the method described by Dewanto et al. (2002) and the results were determined as catechin equivalents (mg/100 g of dry weight). At a concentration of 1 mg/mL, PWE and PWEO were diluted with water (4 mL) in a 10 mL volumetric flask. Initially, 5% NaNO_2_ solution (0.3 mL) was added to each volumetric flask; at 5 min, 10% AlCl_3_ (0.3 mL) was added; and at 6 min, 1.0 mol/L NaOH (2 mL) was added. Water (2.4 mL) was then added to the reaction flask and mixed well. Absorbance of the reaction mixture was read at 510 nm.

### Preparation of SFO samples for oxidative stability determination

PWE and PWEO were added to SFO at 1200 ppm. Synthetic antioxidant (TBHQ) was employed at a limit of 100 ppm to compare the efficacy of natural antioxidants (Fki et al. 2005). All the samples (120 mL each) were placed in dark brown colored reagent bottles with narrow necks, without stoppers, and stored at ambient conditions (25°C for 60 days) (Anwar et al. 2007). Control samples were also placed under the same storage conditions. Samples (20 g) were removed periodically every 0, 15, 30, 45, and 60 days for analysis. Immediately after storage period, oil samples were withdrawn for triplicate analysis. The oils were sampled for each measurement from separate bottles.

### Analytical procedures

Peroxide value (PV) was measured according to the spectrophotometric method of the International Dairy Federation as described by Shantha and Decker (1994). The carbonyl value (CV) of the oils was measured according to the method developed by Endo et al. (2001), using 2‐propanol and 2,4‐decadienal as solvent and standard, respectively. AOCS Official Method Cd 3d‐63 (AOCS 1990) was used to determine the acid value (AV). Total polar compounds (TPC) content was determined according to the economical micromethod developed by Schulte (2004). Oxidative stability index (OSI) is a method whereupon air passes through a lipid under a specific temperature, at which point volatile acids decomposed from lipid peroxidation are driven out by the air and subsequently dissolved in water, thereby increasing its conductivity. The conductivity of the water is constantly measured, and the OSI value is defined as the hours required for the rate of conductivity to reach a predetermined level (Liu et al. 2014).

### Statistical analysis

All analyses were performed in triplicate and data reported as means ± standard deviation (SD). Statistical analyses were conducted using SPSS (Statistical Program for Social Sciences, SPSS Corporation, Chicago, IL) version 18.0 for Windows. Data were subjected to analysis of variance. Significant differences (*P* < 0.05) were calculated using Duncan's multiple range tests.

## Results and Discussion

### TP and TF contents

Phenolic component is one of the major groups of phytochemicals ubiquitously present in the plant kingdom, and they have received much attention in the recent years due to their potential health benefits as an antioxidant and protective agents against cancer and several other diseases (Plumb et al. 1997).

Flavonoids are the most abundant polyphenols in plants, and they have been shown to have potent antioxidant and anticancer activities (Adom and Liu 2002; Dykes and Rooney 2007).

Results of the TP in PWE and PWEO are presented in Table 1. The differences between the content of TP between PWE and PWEO were statistically significant (*P* < 0.05). The PWE gives higher content of TP (966.72 ± 44.27 mg GAE/g sample) than TP of PWEO (355.84 ± 27.53 mg GAE/g sample). Similar results were also observed related to TF (Table 1). These data suggested that PWE is valuable source of phenolic and flavonoid compounds that might be extracted and used in the food and drug industries.

**Table 1:** Total phenolic (TP) and total flavonoid (TF) contents of pussy willow extract and PW essential oil

Extraction	Total phenolic content(mg GAE/g samples ± SD)	Total flavonoid content(mg/100 g samples ± SD)
Pussy willow extract	966.72 ± 44.27^a^	619.45 ± 12.2^a^
Pussy willow essential oil	355.84 ± 27.53^b^	195.45 ± 9.12^b^

Means ± SD (standard deviation) within a column with the same lowercase letters are not significantly different at p<0.05.

A linear correlation between the antioxidant activity and polyphenolic contents of the plant extracts has been reported by other researchers (Luximon‐Ramma et al. 2002; Karimi et al. 2014). Kamkar et al. (2010), which showed that *Mentha pulegium* extracts had more antioxidant activity than *M. pulegium* EO. They stated that the lower antioxidative activity of the *Mentha pulegium* EO can be due to the lack of some of the different antioxidants in the EO.

### Stability of SFOs as affected by the addition of PWE and PWEO

#### Peroxide value and carbonyl value

**Figure 1:**
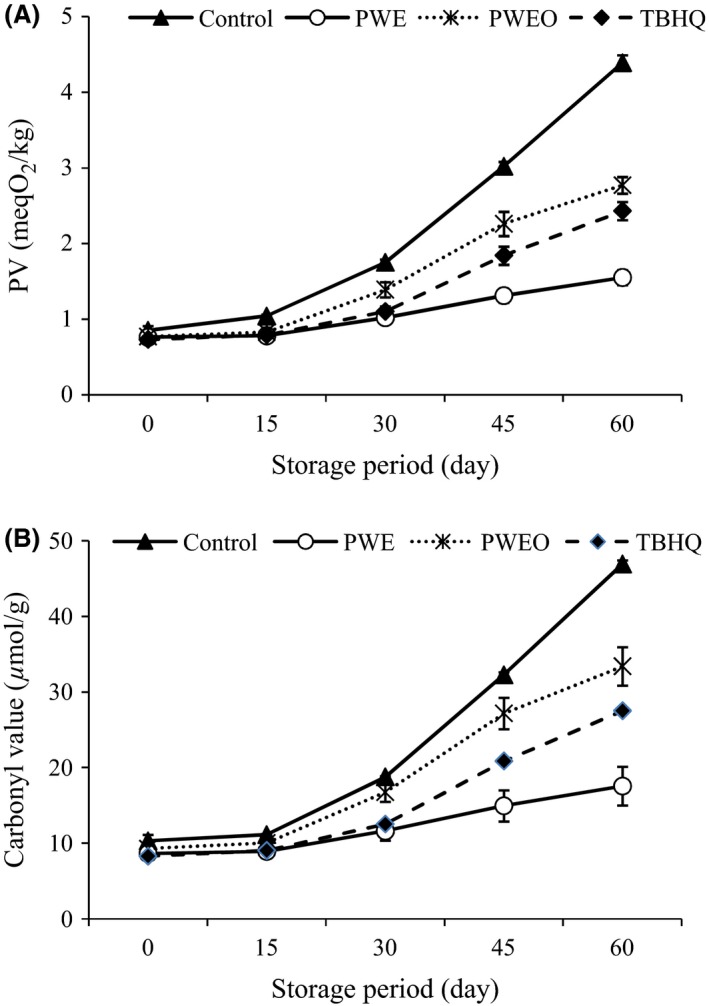
Effect of pussy willow extract (PWE) and PW essential oil (PWEO) on peroxide value (A) and carbonyl value (B) of sunflower oil during ambient storage. Error bars show the variations of three determinations in terms of standard deviation.

PV was used as indicators for the primary oxidation of oil and lipids. Hydroperoxide is the primary oxidation product produced as a result of lipid oxidation (Asnaashari et al. 2015b). The quality of the oil may be decreased if it breaks down into nonvolatile and volatile secondary products (Poiana 2012; Mei et al. 2014). By increasing the storage period time, a continuous increase in PV was observed in all the samples (Fig. 1A). The rise of PV amount was very slow, at the beginning, but it started increasing by 15th day of storage and continued to increase further with the increase in storage period, reaching a maximum value after 60 days storage. A significant difference (*P* < 0.05) in PV was observed between the control and SFO samples containing PWE and PWEO, which slowed the rate of peroxide formation revealing good antioxidant efficacy in stabilizing oil (Ben‐Ali et al. 2014). Highest PV was observed for control followed by SFO containing PWEO, TBHQ, and PWE, respectively. Such pattern was presented by Kamkar et al. (2010) on the methanol extract and EO of *Mentha pulegium* compared to the butylated hydroxytoluene (BHT) when added to SFOs.

According to Woyewoda et al. (1986), primary oxidation products (peroxides) are converted into secondary products that contain carbonyl groups. These compounds are more stable than hydroperoxides and are considered to be a good index of oxidative changes in lipids. A similar trend was also observed about CV in all treatments (Fig. 1B). Thus, the lowest PV and CV were observed in SFO containing PWE. It may be attributed as a higher antioxidant ability of PWE which is related to its polyphenol component. The formation of fatty acid free radicals, which react with or absorb oxygen in the autoxidation process, was prevented by them. This delays the onset of the autoxidative process in oil (Turhan et al. 2009). These results are in agreement with that described previously (Iqbal et al. 2008; Ben‐Ali et al. 2014; Mei et al. 2014).

#### Acid value

**Figure 2:**
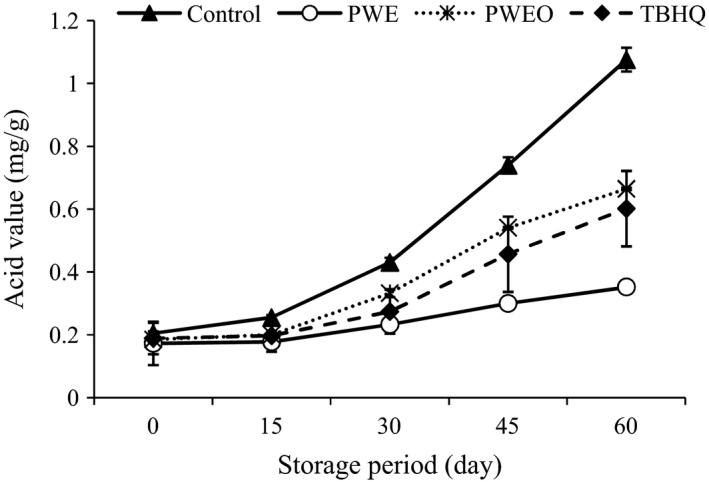
Effect of pussy willow extract (PWE) and PW essential oil (PWEO) on acid value of sunflower oil during ambient storage.

Acid value has become a good indicator of the quality. AV is the *number* that expresses, in *milligrams*, the quantity of KOH (in mg) necessary to neutralize free fatty acids in 1 g of oil (Ban and Kang 2014). AV contents of the SFO as affected by the TBHQ, PWE, and PWEO during ambient condition are given in Figure 2. By increasing the storage period, a continuous increase in AV was observed in all the samples. The steady increase in the AV can be attributed partly to hydrolysis of triacylglycerols and partly to the component carboxyl groups present in autoxidative process (Petukhov et al. 1999; Nasirullah 2001). Control exhibited the highest AV at all the stages of analyses during storage. There was no statistically significant difference between SFOs that contain PWEO and TBHQ (*P* > 0.05). The oil samples stabilized with PWE showed lowest levels of AV compared to the other, which suggests the superiority of the PWE over the other antioxidants, such as PWEO and TBHQ, in delaying oxidation in SFO.

### Total polar compounds

**Figure 3:**
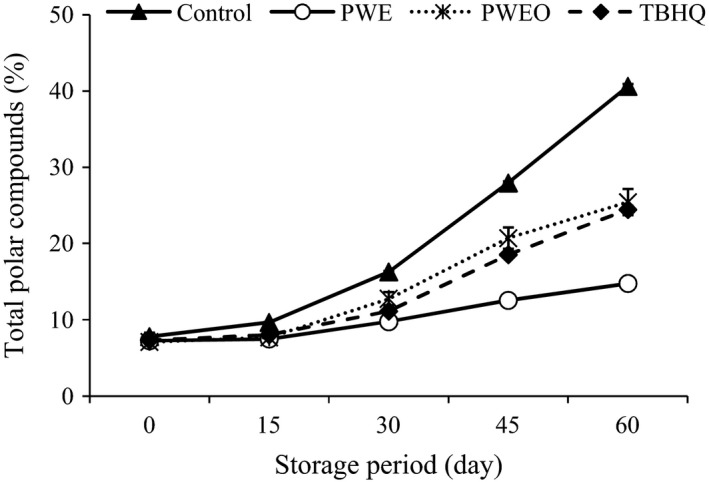
Effect of pussy willow extract (PWE) and PW essential oil (PWEO) on total polar compounds of sunflower oil during ambient storage.

Polar compounds is an important indicator of oil quality of used oils, giving information of the total amount of newly formed compounds having higher polarity than that of triacylglycerols (Li et al. 2015). The TPC of treatments during the ambient storage are presented in Figure 3. The TPC content increased until the end of the storage period (in all samples). The TPC content of SFO contain antioxidants significantly (*P* < 0.05) lower than the control samples during the storage time. Literature has shown that the fractions of TPC isolated from oxidized oils are toxic to laboratory animals (Pantzaris 1998). Therefore, it has been purposed that frying oils containing more than 24–27% of TPC content should be discarded (Firestone 1993). Only the control among all the samples reached the discarding range of TPC content during storage condition.

The lowest TPC content at the end of storage period was observed in SFO containing PWE. Farhoosh and Kenari (2009) stated that canola oils containing sesame and rice bran oils were more stable than the canola oils without additives during frying, based on TPC formation.

### Oxidative stability index

**Figure 4:**
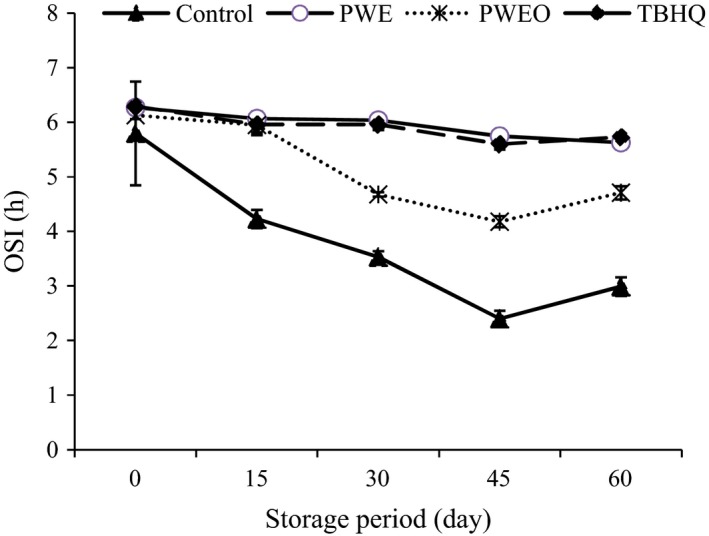
Effect of pussy willow extract (PWE) and PW essential oil (PWEO) on oxidative stability index of sunflower oil during ambient storage.

The OSI is an American Oil Chemists' Society approved method that determines the relative resistance of oils to oxidation. Oxidative stability instrument can be employed to determine OSI of oil samples and it is the commercial piece of equipment for the automated determination of oxidative resistance as well as it is well‐known by the fats and oils industries (Shahidi and Zhong 2005; Senanayake 2013). Changes in the OSI of the SFO affected by the PWE, PWEO, and TBHQ during the ambient storage are shown in Figure 4. A continued decrease in OSI with the increase in storage period was observed in all the samples. But in control, the rate of decrease was higher than others. There was no statistically significant difference between SFOs containing PWE and TBHQ (*P* > 0.05). The rate of decrease in OSI throughout the storage period indicates higher efficiency of PWE for the stabilization of SFO than PWEO.

## Conclusion

The results of the present study apparently demonstrated that the PWE had high TP and TF contents. Moreover, PWE exhibited strong antioxidant activity in stabilizing SFO during ambient storage, which was almost more effective than the antioxidant activity of synthetic antioxidants (TBHQ) as well as PWEO. Therefore, it is suggested that the PWE could be safely used for the stabilization of food systems instead of synthetic antioxidants.

## Conflict of Interest

None declared.
